# Direct and indirect regulation of bone metabolism by lactoferrin

**DOI:** 10.3389/fendo.2025.1660312

**Published:** 2025-10-15

**Authors:** Yangyang Wu, Cunxin Zhang, Chaoliang Lv

**Affiliations:** ^1^ Clinical Medical College of Shandong Second Medical University, Weifang, China; ^2^ Spine Surgery Department of Jining First People's Hospital, Jining, China

**Keywords:** lactoferrin, bone metabolism, osteoblasts, osteoclasts, osteoporosis

## Abstract

Lactoferrin exerts positive regulation on bone metabolism through both direct and indirect pathways. Directly, it modulates osteoblasts, osteoclasts, and chondrocytes via factors such as insulin-like growth factor (IGF), low-density lipoprotein receptor-associated protein (LRP), transforming growth factor β (TGF-β) receptor, and bone morphogenetic proteins (BMPs). These factors promote differentiation and inhibit apoptosis of bone metabolism-related cells through signaling pathways including the receptor activator of nuclear factor kappa-B (RANK), its ligand RANKL, and osteoprotegerin (OPG), nuclear factor kappa-light-chain-enhancer of activated B cells (NF-κB), mitogen-activated protein kinase/extracellular signal-regulated kinase (MAPK/ERK), macrophage colony-stimulating factor (M-CSF), Ca2+, phosphoinositide 3-kinase/protein kinase B (PI3K/Akt), Wnt/β-catenin, BMP-2/Smad, and TGF-β. Indirectly, lactoferrin influences skeletal muscle, energy metabolism, intestinal microbiota, immune function, and calcium-phosphorus homeostasis, all of which positively affect bone metabolism. In-depth research into lactoferrin-derived peptides and their complexes for slow-release systems may open new avenues for treating orthopedic diseases. However, the mechanisms by which lactoferrin regulates bone metabolism remain incompletely understood. This review aims to summarize these mechanisms and highlight recent advances in lactoferrin-derived peptides and their complexed slow-release systems, providing a comprehensive basis for exploring lactoferrin as a potential therapeutic target in bone diseases.

## 1 Preface

As the global aging problem is becoming increasingly serious, the prevalence of osteoporosis in the world’s total population is reported in the relevant literature to be 18.3%, with about 23.1% in women and 11.7% in men, and osteoporosis has become an important chronic disease that seriously jeopardizes human society (1). Abnormal bone metabolism is an important cause of osteoporosis (2). Currently, studies on the regulation of bone metabolism are becoming more and more in-depth, and some studies have shown that lactoferrin (LTF) plays an important role in the regulation of bone metabolism (3). Lactoferrin is an endogenous multifunctional iron-binding glycoprotein with a variety of biological functions such as immunomodulation, metabolic regulation, bone homeostasis regulation, antiviral, antihypertension (4), attenuation of cardiac remodeling and improvement of cardiac function, which has potential therapeutic value for a variety of diseases, such as osteoporosis, osteoarthritis, bone fracture, obesity, hypertension, and Alzheimer’s (5). Lactoferrin can directly regulate bone formation and resorption in both directions by promoting osteoblast differentiation and mineralization and inhibiting osteoclast proliferation and differentiation. Not only that, the indirect regulation of other systems by lactoferrin also has a positive effect on bone-related cells, such as lactoferrin through the regulation of skeletal muscle, glucose metabolism, and intestinal microorganisms (6–8). In view of the powerful bone metabolism regulation function of lactoferrin, there is a growing interest in the role of lactoferrin in the regulation of bone metabolism.

## 2 Overview of lactoferrin

### 2.1 Sources of lactoferrin

Lactoferrin, first isolated and purified from human and bovine milk in 1960, is a single-chain polypeptide glycoprotein with a molecular weight of approximately 78 kDa and a highly flexible structure (9). Lactoferrin is an endogenous multifunctional protein that is widely found in a variety of fluids such as human milk, cow’s milk, human saliva and semen, as well as on the surfaces of mucous membranes and in the secondary granules of some neutrophils (10). Among them, lactoferrin located in body fluids and mucosal surfaces is the secreted type, which is mainly involved in defense against microbial infections, whereas lactoferrin located in secondary granules of neutrophils has additional immunomodulatory effects (11). Lactoferrin is categorized as a member of the transferrin family due to its structural similarity to serum transferrin, which has the ability to bind iron ions reversibly (12). The wide distribution and delicate structure of lactoferrin facilitates its diverse functions.

### 2.2 Functions of lactoferrin

Lactoferrin has a wide range of physiological functions, including immunomodulation (13), antiviral, antioxidant (14, 15), anticancer (16), iron metabolism modulation, enhancement of early neurodevelopment and cognitive function, modulation of intestinal flora homeostasis, modulation of lipid metabolism, antihypertensive (17), and cardioprotective (18) effects. Due to its powerful functions, lactoferrin has been widely used in infant milk powder, nutritional and health care products and other fields (19). Not only that, it has been shown in some studies that lactoferrin has an important regulatory effect on bone metabolism, which regulates the functions of osteoblasts, osteoclasts, and chondrocytes directly or indirectly through a variety of pathways, and then positively promotes bone health.

## 3 Overview of bone metabolism

Bone metabolism is a complex metabolic state maintained by bone through the intercellular association of osteoblasts, osteoclasts and osteoclasts (20). Osteoblasts are responsible for bone formation and can secrete a variety of hormones and cytokines (21), and regulate bone formation through signaling pathways such as Wnt/β-catenin, BMP-2/smad, and PI3K/AKT (22). Osteoclasts are multinucleated cells, mainly regulated by RANKL and MCSF, which mediate bone resorption through various signaling pathways (23). Osteoblasts are derived from osteoblasts and can regulate osteoblast and osteoclast activity (24). New studies continue to show that osteoblasts also act at other levels of the body such as energy metabolism, gonadal function, calcium and phosphorus metabolism, the nervous system, and glucose homeostasis through a variety of pathways (20). These studies also express to us that this broad regulation of the function of other systems is not unidirectional, and that these same systems can, in turn, have a critical impact on bone cells. This provides us with a broader picture of the search for targets that act on bone metabolism.

## 4 Lactoferrin regulates bone-associated cells and influences bone metabolism

### 4.1 Mechanisms of lactoferrin regulation of osteoblasts

Insulin-like growth factor 1 (IGF-1) is the most abundant growth factor in the bone matrix, plays a crucial role in bone growth and maintenance, and can be regulated by multiple factors (2, 25). IGF-1 in the bone matrix during bone remodeling stimulates the differentiation of recruited mesenchymal stem cells (MSCs) to osteoblasts through activation of mammalian target of rapamycin (mTOR) (26). Lactoferrin can enhance osteoblast proliferation and effectively inhibit osteoblast apoptosis by upregulating IGF-1 in osteoblasts and thereby stimulating IGF-1R, through activation of the downstream PI3k/Akt/mTOR pathway (27). It has been shown that lactoferrin improves osteoblast proliferation by enhancing IGF-1 signaling mainly by promoting the expression of osteoblast markers, including alkaline phosphatase (ALP) activity, Igf1, osteoglialin (Bglap), and osteoprotegerin/nuclear factor receptor activator of κB ligand (Opg/Rankl) mRNA. not only this, but lactoferrin can also slow down osteoblast senescence by reducing the expression of p16 and p21 through the IGF-1 signaling pathway (28). Low-density lipoprotein receptor-related protein 1 (LRP1) is an endocytosed transmembrane receptor involved in a variety of biological pathways, which not only serves as a signaling receptor, but also has a variety of functions such as ligand endocytosis, inhibition of cancer cell growth and metastasis, and regulation of the diffusion of microtubule-associated proteins in the brain (29, 30). Lactoferrin can activate the extracellular signal-regulated kinase pathway (ERK pathway) in osteoblasts through LRP1, and the activation of this pathway can promote osteoblast differentiation and bone formation. Lactoferrin also promotes bone anabolism through mitogenic signaling from LRP1 to p42/44 MAPK (31). In addition, lactoferrin inhibits osteoblast apoptosis through the LRP1-independent PI3K/Akt pathway, thus acting as an osteoclast protector (32). Both classical and non-classical TGF-β signaling pathways are involved in lactoferrin-mediated osteogenic activity in C3H10T1/2 cells (33), and lactoferrin can bind to TGF-β receptor II and activate the classical TGF-β signaling pathway, which results in the up-regulation of osteogenic genes such as *Runx2*, osterix and type I collagen. Also lactoferrin induced phosphorylation of ERK1/2 in C3H10T1/2 cells, suggesting activation of the non-classical TGF-β signaling pathway. Dorit et al. demonstrated that lactoferrin promotes osteoblast proliferation by enhancing the activity of prostaglandin-endoperoxide synthase 2 (Ptgs2) and nuclear factor of activated T-cell transcription factor (NFATc1). Similarly, inhibition of COX2 or NFATc1 activity blocked the mitogenic effects of lactoferrin in osteoblasts (34). Lactoferrin was found to upregulate vitamin D receptors in osteoblasts and promote osteoblast activity, which in turn improved bone density in vitamin D-deficient mice (35). Lactoferrin also plays a role in regulating lncRNA expression in osteogenic differentiation of mesenchymal stem cells (36). It has been shown that lactoferrin induces the synthesis of vascular endothelial growth factor VEGF and fibroblast growth factor-2FGF2 in MC3T3-E1 cells in a p44/p42MAP kinase-dependent manner, further promoting bone formation (37). Lactoferrin further enhances Beclin1-dependent autophagy activation by inhibiting BCL2 expression in osteoblasts, which in turn enhances osteoclastogenesis (38).Prof. Inubushi T found that lactoferrin was able to play an osteogenic role by activating the Smad2/3 and p38 MAPK signaling pathways (39).

The mechanisms by which lactoferrin regulates osteoblasts are complex and varied, and although relevant studies have demonstrated that lactoferrin can play a role in promoting bone formation through the factors and signaling pathways mentioned above, deeper mechanistic studies are still the focus of subsequent research.

**Figure 1:**
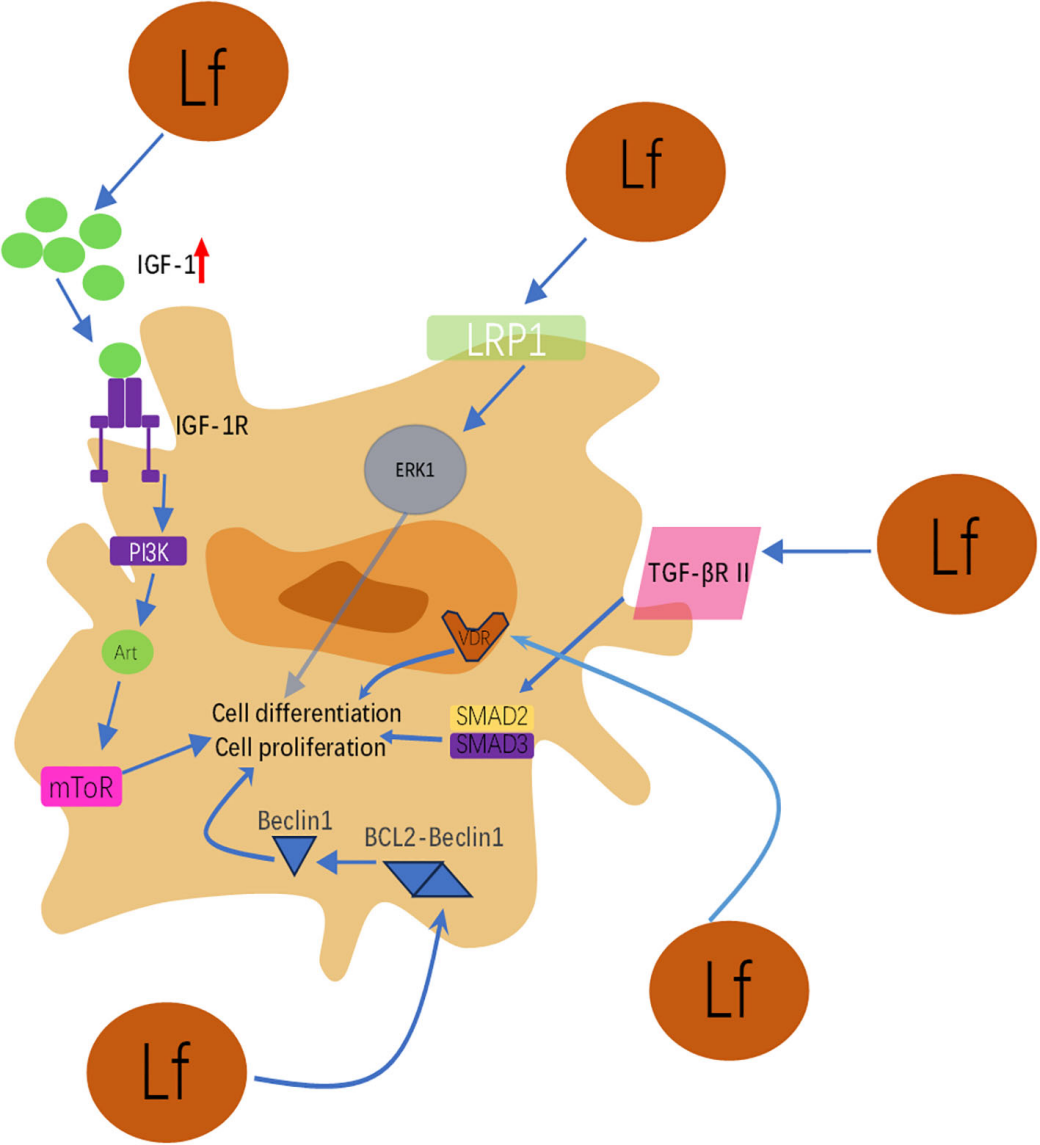
Schematic diagram of lactoferrin’s multi-pathway regulation of osteoblast function.

This schematic integrates the molecular mechanisms by which lactoferrin (Lf) promotes osteoblast proliferation, differentiation, survival, and inhibits apoptosis through activation of multiple key signaling pathways. As illustrated, its action primarily involves the following pathways: (1) IGF-1/PI3K/Akt/mTOR pathway: Lactoferrin activates the insulin-like growth factor-1 (IGF-1) receptor (IGF-1R) and downstream PI3K, Akt, and mTOR signaling cascades by upregulating IGF-1 expression in osteoblasts. This pathway is one of the core mechanisms by which lactoferrin promotes osteoblast proliferation and differentiation while inhibiting apoptosis. It also delays cellular senescence by reducing p16 and p21 expression. (2) LRP1/ERK pathway: Lactoferrin binds as a ligand to low-density lipoprotein receptor-related protein 1 (LRP1), thereby activating extracellular signal-regulated kinase (ERK1/2). Activation of the ERK pathway is crucial for driving osteoblast differentiation and bone formation. (3) TGF-β signaling pathway: Lactoferrin directly binds to transforming growth factor-β receptor II (TGF-βR II), activating both the canonical Smad2/3 pathway and the non-canonical ERK pathway. This dual activation synergistically upregulates key osteogenic genes such as Runx2, Osterix, and type I collagen, enhancing osteogenic activity. (4) Vitamin D Receptor (VDR) Upregulation: Lactoferrin enhances VDR expression in osteoblasts, thereby amplifying vitamin D signaling and improving osteoblast function and bone density under vitamin D deficiency conditions.(5) Autophagy Activation and Angiogenic Factor Induction: Lactoferrin promotes osteogenic differentiation by inhibiting BCL2 expression and enhancing Beclin1-dependent autophagy activity. Concurrently, it induces synthesis of vascular endothelial growth factor (VEGF) and fibroblast growth factor-2 (FGF2) via the p44/p42 MAPK pathway, creating a favorable microenvironment rich in vasculature and growth factors for bone formation. Summary: **Figure 1** clearly demonstrates that lactoferrin does not act through a single pathway but functions as a multi-target modulator, synergistically regulating a complex signaling network to collectively promote bone formation. This multifaceted action endows it with significant potential for treating bone metabolic disorders such as osteoporosis.

### 4.2 Mechanisms of lactoferrin regulation of osteoclasts

Lactoferrin inhibits the NF-κB signaling pathway in osteoblasts and reduces RANKL production, which in turn inhibits osteoclastogenesis (40). It has been shown that lactoferrin has the ability to inhibit lipopolysaccharide-induced osteoclastogenesis (41). Lactoferrin inhibits TNF-α formation by inhibiting NF-κB and mitogen-activated protein kinase pathways, which further inhibits osteoclasts value-added (42). In addition, lactoferrin can limit osteoclastogenesis by scavenging ROS associated with osteoclastogenesis and thus limiting osteoclastogenesis (43). In conclusion, lactoferrin regulation of osteoclasts inhibits bone resorption at levels maintaining bone health.

### 4.3 Mechanisms of lactoferrin regulation of chondrocytes

Lactoferrin was shown to significantly inhibit IL-1β-induced chondrocyte apoptosis (44). Lactoferrin inhibits dexamethasone-induced chondrocyte injury by up-regulating extracellular signal-regulated kinase 1/2 (ERK1/2) and down-regulating proteins involved in apoptosis, such as FASL, FAS, and Caspase3, thus exerting a chondrocyte-protective function (45). Lactoferrin inhibited the expression of N-calmodulin, collagen X, and ALP in the ATDC5 chondrogenic progenitor cell line, which in turn inhibited its overdifferentiation causing joint diseases. In addition, the expression of Sox9 as well as Smad2/3 was increased in the ATDC5 chondrogenitor cell line in the presence of lactoferrin, suggesting that lactoferrin regulates cartilage differentiation by upregulating the Smad2/3-Sox9 signaling pathway (46). Lactoferrin is also able to enhance articular cartilage repair by activating the ERK signaling pathway in chondrocytes, which in turn enhances BMP7 expression (46). The protective effect of lactoferrin on chondrocytes makes it a potential new target for the treatment of articular cartilage damage.

### 4.4 Context-dependent regulation of signaling pathways by lactoferrin

The seemingly contradictory effects of lactoferrin on the NF-κB and ERK signaling pathways—inhibition of the former with simultaneous activation of the latter—may appear contradictory at first glance. However, this contradiction highlights the contextual and cell-specific nature of lactoferrin’s regulatory effects. NF-κB inhibition occurs mainly in osteoclasts and their precursors and leads to the suppression of excessive bone resorption and the formation of inflammatory osteoclasts. In contrast, activation of the ERK signaling pathway is observed mainly in osteoblasts and chondrocytes, where it promotes cell proliferation, differentiation, and survival, thereby improving bone formation and repair. This dual modulation demonstrates lactoferrin’s ability to simultaneously reduce bone loss and stimulate bone accumulation through different mechanisms in different cell types, rather than representing a mechanical inconsistency. In addition, Lactoferrin’s capacity to interact with both standard (Smad2/3-based) and non-standard (ERK1/2-based) TGF-β signaling pathways demonstrates its comprehensive stimulatory effect on this crucial bone signaling system. This dual activation is not contradictory; rather, it highlights lactoferrin’s synergistic mechanism of simultaneously enhancing bone gene expression (via Smad) and improving cell proliferation and survival (via ERK), which together promote robust bone formation. The direct regulatory mechanisms of lactoferrin on bone metabolism are summarized in **Table 1**.

**Table 1:** Direct regulatory mechanisms of lactoferrin on bone-related cells.

Cell type	Mechanism of action (detailed description)	Key signaling pathway/molecule	Biological effect	Key references
Osteoblasts	1. Lactoferrin upregulates the expression of Insulin-like Growth Factor-1 (IGF-1) in osteoblasts, activating the IGF-1R receptor and its downstream PI3K/Akt/mTOR signaling pathway.	IGF-1/PI3K/Akt/mTOR	Promotes osteoblast proliferation and differentiation while inhibiting apoptosis. Concurrently reduces the expression of senescence markers p16 and p21, delaying cellular senescence.	(27, 28)
	2. Lactoferrin binds to the Low-density Lipoprotein Receptor-related Protein 1 (LRP1), thereby activating the Extracellular Signal-regulated Kinase (ERK1/2) pathway.	LRP1/ERK	Promotes osteoblast differentiation and enhances bone formation.	(31)
	3. Lactoferrin directly binds to the Transforming Growth Factor-beta (TGF-β) Receptor II, activating both the canonical Smad2/3 signaling pathway and the non-canonical ERK pathway.	TGF-β/Smad, ERK	Synergistically upregulates the expression of key osteogenic genes such as *Runx2*, Osterix, and Type I collagen, enhancing osteogenic activity.	(33)
	4. Lactoferrin upregulates the expression of the Vitamin D Receptor (VDR) in osteoblasts.	VDR	Potentiates Vitamin D signaling, improving osteoblast activity and bone density under Vitamin D-deficient conditions.	(35)
	5. Lactoferrin enhances Beclin1-dependent autophagy by inhibiting BCL2 expression and induces the synthesis of Vascular Endothelial Growth Factor (VEGF) and Fibroblast Growth Factor-2 (FGF2) in a p44/p42 MAPK-dependent manner.	Beclin1 (autophagy), VEGF, FGF2 (MAPK)	Autophagy activation promotes osteogenic differentiation; the production of VEGF and FGF2 stimulates angiogenesis, creating a favorable microenvironment for bone formation.	(37, 38)
Osteoclasts	1. Lactoferrin inhibits the Nuclear Factor-kappa B (NF-κB) and Mitogen-Activated Protein Kinase (MAPK) pathways, reducing the production of the Receptor Activator of Nuclear Factor-kappa B Ligand (RANKL).	NF-κB, MAPK	Inhibits Lipopolysaccharide (LPS)- or Tumor Necrosis Factor-alpha (TNF-α)-induced osteoclastogenesis, thereby suppressing osteoclast formation and proliferation.	(40–42)
	2. Lactoferrin scavenges Reactive Oxygen Species (ROS) associated with osteoclastogenesis.	Reactive Oxygen Species (ROS)	Limits osteoclast differentiation through its antioxidant effects, thereby inhibiting bone resorption.	(43)
Chondrocytes	1. Lactoferrin activates the AKT1/CREB1 signaling pathway.	AKT1/CREB1	Inhibits Interleukin-1 beta (IL-1β)-induced chondrocyte apoptosis, exerting a chondroprotective effect.	(44)
	2. Lactoferrin upregulates the phosphorylation of ERK1/2 and downregulates the expression of apoptosis-related proteins FASL, FAS, and Caspase-3.	ERK1/2	Protects chondrocytes from dexamethasone-induced injury.	(45)
	3. Lactoferrin downregulates the expression of N-cadherin, Collagen X, and Alkaline Phosphatase (ALP), while upregulating the expression of Sox9 and Smad2/3.	Sox9, Smad2/3	Inhibits the excessive hypertrophic differentiation of ATDC5 chondroprogenitor cells, maintaining a stable chondrocyte phenotype and preventing joint diseases.	(46)
	4. Lactoferrin activates the Extracellular Signal-regulated Kinase (ERK) pathway.	ERK → BMP7	Enhances the expression of Bone Morphogenetic Protein 7 (BMP7), thereby promoting the repair of articular cartilage.	(46)

## 5 Other mechanisms by which lactoferrin regulates bone metabolism/homeostasis

### 5.1 Lactoferrin modulation of skeletal muscle affects bone metabolism

Lactoferrin not only directly regulates bone metabolism-related cells such as osteoblasts and osteoclasts, but also indirectly influences bone metabolism through skeletal muscle.

Skeletal muscle is capable of secreting a variety of growth factors and cytokines, which can regulate the function of skeletal cells and thus affect bone metabolism (47, 48). Irisin, a myogenic factor, has been shown to regulate bone metabolism through a variety of signaling pathways: Irisin promotes osteoblast precursor cell differentiation to osteoblasts through the Wnt/β-catenin signaling pathway, and activates the p38MAPK/ERK signaling pathway to enhance osteoclast value-addition, differentiation and mineralization. In addition, irisin down-regulates the expression of the senescence marker P21 in osteoblasts (49). Some findings have shown that lack of lactoferrin impairs satellite cell (SC) proliferation and skeletal muscle regeneration (6). Therefore, lactoferrin functions to influence bone metabolism through the regulation of skeletal muscle, followed by the regulation of related myogenic factors such as irisin, FGF2, and OGN. Lactoferrin regulation of satellite cells (SCs) proliferation may be dependent on the activation of the ERK pathway, which, upon activation, promotes the cell cycle from the G1 phase into the S phase by inducing the assembly of cytokinin D and the cytokinin D-CDK4 complex (50). The act of maintaining a certain level of cell cycle protein D in the G1 phase and thus allowing the cell to enter the S phase is inextricably linked to the sustained activation of the ERK pathway (51). In addition, a significant decrease in the levels of p-ERK1/2, cyclin D, and CDK4 was observed in mice deficient in lactoferrin.

### 5.2 Lactoferrin modulation of energy metabolism affects bone metabolism

Beyond regulating bone metabolism through skeletal muscle, lactoferrin’s role in controlling systemic energy metabolism can further influence bone homeostasis.

Glucose plays a crucial role in skeletal homeostasis (52, 53). Lactoferrin for blood glucose levels and insulin activity can be regulated in several ways (54). Lactoferrin promotes glucose transport to small intestinal epithelial cells by down-regulating Ca2+ and cAMP signaling pathways, which in turn exerts a hypoglycemic effect, an effect that may be reached through sodium-dependent glucose transporter protein (SCLT) 1 (7, 55). Lactoferrin enhances insulin signaling through a PPARγ-dependent cascade reaction and also functions as an insulin sensitizer by upregulating hepatic glucokinase, the rate-limiting enzyme of glycolysis, and by stimulating hepatic glucose uptake (56). Lactoferrin has sugar-binding properties that allow it to bind to glucose and other sugars through multiple hydrogen bonds and van der Waals interactions (57), a property that may modulate free sugars in the gut and thus function as a regulator of glucose metabolism. Lactoferrin may also have the function of inhibiting the activation of the corticosterone axis, which leads to the improvement of insulin resistance (58). Lactoferrin can exert its hypoglycemic function by maintaining insulin secretion through upregulation of GLP-1 (59). In mouse osteoblast cultures, insulin promotes 14C glucose uptake and oxidation in a dose-dependent manner, which in turn promotes osteoblast differentiation. Serum levels of osteocalcin (OC) secreted by osteoblasts were significantly higher in diabetic patients with good glycemic control, two observations that indirectly show the association of osteoblasts with overall glucose homeostasis (60, 61), suggesting that lactoferrin stabilizes bone formation by lowering blood glucose levels.

### 5.3 Lactoferrin modulates gut microbes to influence bone metabolism

Gut microbes are diverse over 1000 species (62) and are associated with energy metabolism, nutrient supply, immune regulation, inflammatory response and a wide range of diseases. However, it has been shown that gut microbes have an important role in regulating bone metabolic processes (63).

Bovine lactoferrin promotes the proliferation of human intestinal epithelial cells by activating the PI3K/Akt signaling pathway (8). The increase in the number of mature cells in the intestinal epithelium increases the area of nutrient absorption and enhances the intestinal nutrient absorption capacity, which in turn provides a more suitable growth environment and growth space for intestinal microorganisms (64). The regulation of bone metabolism by gut microorganisms is bi-directional, which both promotes bone formation and enhances bone resorption. Novince et al. found that gut microorganisms can enhance osteoclast activity by regulating the RANKL/OPG ratio (65). However, Bifidobacterium bifidum can down-regulate the RANKL/OPG ratio to attenuate osteoclast activation, and it can also up-regulate the expression of Bmp-2 and Sparc genes *in vivo*, which promote osteoblast differentiation, and Sparc gene expression promotes bone mineralization (66). Some studies have found that spore-forming bacteria in gut microbes catalyze intestinal 5-HT production, and its elevation negatively regulates bone mass (67). However, it has also been found that the gut microorganism Lactobacillus can enhance osteoblast proliferation by elevating insulin-like growth factor (IGF-1) levels and thereby enhancing osteoblast proliferation (68). In addition, Lactobacillus Royale reduces Trap5 expression and RANKL reactivation, decreases T-lymphocyte levels, inhibits TNF and Wnt10 production, and maintains skeletal homeostasis by inhibiting osteoclast activity by these means (69). Compared with piglets fed regular whole milk, piglets fed bovine transgenic recombinant human lactoferrin showed increased concentrations of Bifidobacterium bifidum and Lactobacillus lactis and decreased concentrations of Salmonella and Escherichia coli (70), suggesting that lactoferrin positively regulates the distribution of the intestinal flora to enhance the diversity of microorganisms in the intestinal tract, thereby enhancing the role of the intestinal flora in the regulation of bone metabolism. regulation of bone metabolism by the intestinal flora.

This schematic diagram summarizes the synergistic mechanism by which lactoferrin (Lf) indirectly promotes osteoblast differentiation and bone formation by regulating distal systems such as skeletal muscle, energy metabolism, and the gut microbiota. Its primary pathways are as follows: (1) Skeletal Muscle-Myokine Pathway: Lactoferrin activates the ERK signaling pathway in muscle satellite cells, promoting their proliferation and muscle regeneration. Functionally enhanced skeletal muscle secretes increased levels of myokines such as irisin. Irisin has been demonstrated to promote osteoblast proliferation, differentiation, and mineralization by activating the p38 MAPK/ERK pathway. (2)Gut Microbiota-Immune/Metabolic Pathway: Lactoferrin significantly improves gut microbiota composition by increasing beneficial bacteria (e.g., Bifidobacterium, Lactobacillus) and reducing harmful bacteria (e.g., Salmonella). This microbiota modulation confers multiple benefits: It lowers levels of receptor activator of nuclear factor kappa-B ligand (RANKL), thereby inhibiting osteoclast activity. It elevates systemic insulin-like growth factor-1 (IGF-1) levels, directly promoting osteoblast proliferation. (3) Energy Metabolism-Insulin Pathway: Lactoferrin promotes insulin secretion by upregulating glucagon-like peptide-1 (GLP-1). Insulin not only maintains blood glucose stability but also directly stimulates osteoblast glucose uptake and utilization in a dose-dependent manner, thereby supporting their differentiation and function.

Summary: As illustrated, factors influenced by lactoferrin—including irisin, IGF-1, and insulin—collectively act on osteoprogenitor cells, ultimately converging on osteoblast differentiation and activation. This clearly demonstrates that lactoferrin coordinates functions across multiple organ systems—muscle, gut, and energy metabolism—to collectively establish a systemic microenvironment conducive to bone formation.

### 5.4 Lactoferrin regulation of calcium and phosphorus metabolism affects bone metabolism

**Figure 2:**
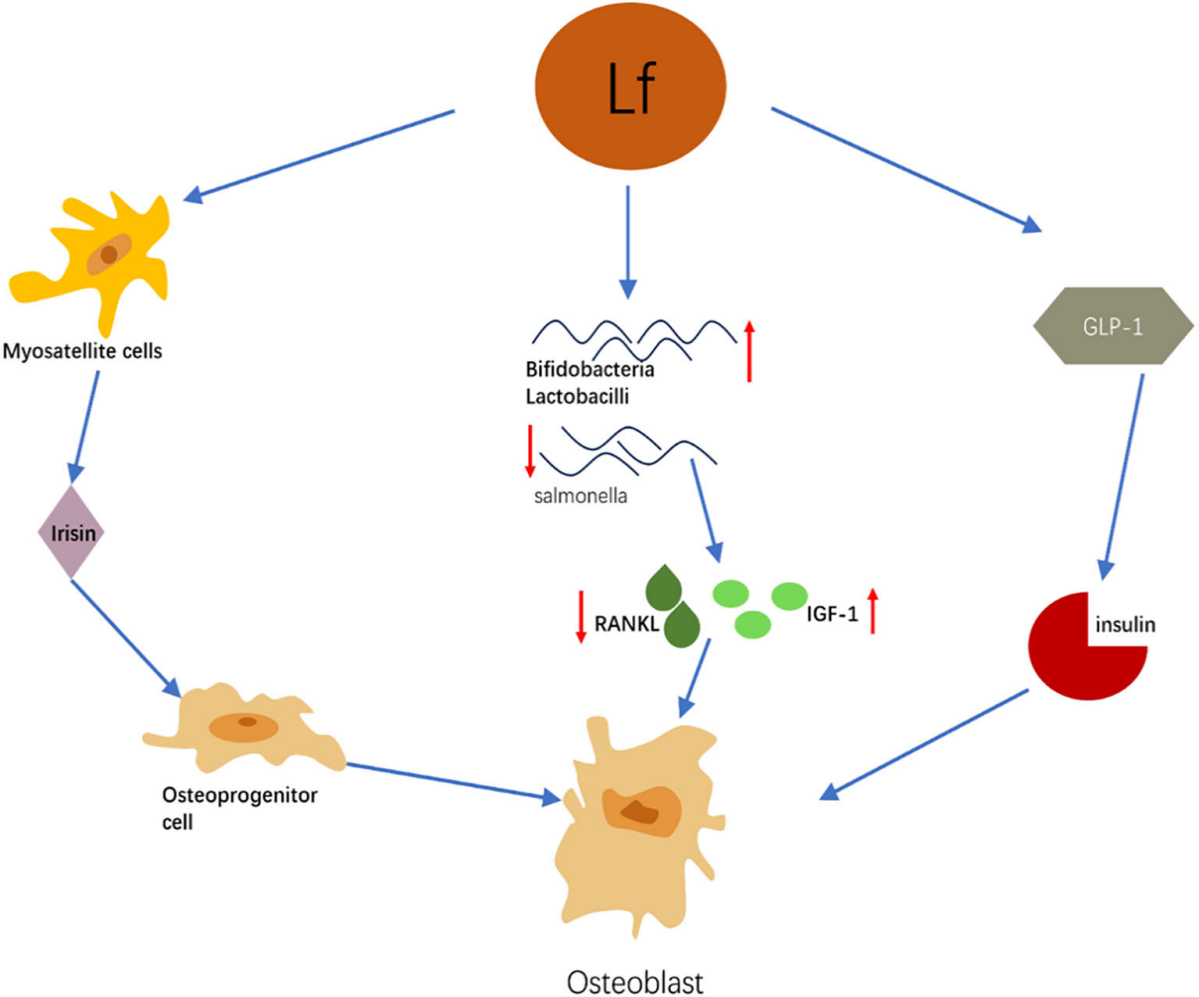
Schematic diagram of the mechanism by which lactoferrin indirectly regulates bone metabolism through multiple systems.

Lactoferrin has sugar-binding properties that enable it to bind to calcium and phosphorus ions in the intestine, forming a stable complex. This complex improves the solubility of minerals in the gut, thereby facilitating their absorption, a process that provides the calcium and phosphorus supply needed for bone mineralization (71). During fracture healing, lactoferrin accelerates new bone formation and mineralization by promoting osteoblast differentiation and function (72). This contributes to shorter fracture healing time and improved healing quality. Lactoferrin also improves bone microstructure, such as increasing the number and thickness of bone trabeculae and improving the connectivity and stability of bone tissue (73). This further enhances the mechanical properties of bone. The mechanism by which lactoferrin indirectly regulates bone metabolism through multiple systems is illustrated in **Figure 2**. The indirect regulatory mechanisms of lactoferrin on bone metabolism are summarized in **Table 2**.

**Table 2:** Mechanisms of lactoferrin in indirectly regulating bone metabolism.

Pathway of action	Mechanism of action (detailed description)	Key factors/pathways	Effect on bone metabolism	Key references
Skeletal Muscle Regulation	1. Lactoferrin activates the ERK signaling pathway in skeletal muscle satellite cells, inducing the assembly of the Cyclin D and CDK4 complex.	ERK → Cyclin D/CDK4	Promotes the transition of satellite cells from the G1 to the S phase, enhancing their proliferation and skeletal muscle regeneration capacity. This increases the secretion of muscle-derived myokines, which indirectly promote osteogenesis.	(6, 50, 51)
	2. By promoting skeletal muscle regeneration and function, lactoferrin increases the secretion of the myokine Irisin.	Irisin → Wnt/β-catenin, p38MAPK/ERK	Irisin promotes the differentiation of osteoblast precursors into osteoblasts and enhances their mineralization capacity by activating the Wnt/β-catenin and p38MAPK/ERK pathways, while simultaneously inhibiting bone resorption.	(49)
Energy Metabolism Regulation	1. Lactoferrin downregulates the Ca^2+^ and cAMP signaling pathways in intestinal cells, thereby enhancing the function of the sodium-dependent glucose transporter (SGLT1).	Ca^2+^/cAMP → SGLT1; PPARγ	Promotes glucose transport into intestinal epithelial cells, exerting a hypoglycemic effect. It also enhances insulin signaling via a PPARγ-dependent cascade, improving the overall metabolic environment to support osteoblast function.	(7, 55, 56)
	2. Leveraging its sugar-binding properties, lactoferrin directly binds to glucose in the gut via hydrogen bonds and van der Waals forces.	Hydrogen bonds/van der Waals forces	Modulates free sugar levels in the intestine, contributing to the maintenance of blood glucose stability, which indirectly provides a stable physiological environment for bone metabolism.	(57)
	3. Lactoferrin inhibits the activation of the hypothalamic-pituitary-adrenal (HPA) axis, reducing corticosterone levels.	Inhibition of corticosterone axis	Ameliorates insulin resistance induced by high corticosterone levels, indirectly promoting bone formation.	(58)
	4. Lactoferrin upregulates the levels of Glucagon-like peptide-1 (GLP-1).	GLP-1 → Insulin	Stimulates insulin secretion, helping to maintain blood glucose stability. Insulin promotes glucose uptake and utilization by osteoblasts in a dose-dependent manner, thereby supporting their differentiation and function.	(59, 60)
Gut Microbiota Regulation	1. Lactoferrin promotes the proliferation of intestinal epithelial cells by activating the PI3K/Akt signaling pathway.	PI3K/Akt pathway	Increases the number of mature intestinal epithelial cells, expanding the nutrient absorption area and improving the growth environment for gut microbiota, which indirectly influences bone metabolism.	(8)
	2. Lactoferrin increases the proportion of beneficial bacteria (e.g., *Bifidobacterium*, *Lactobacillus*) and decreases pathogenic bacteria (e.g., *Salmonella*, *E. coli*).	Probiotics (e.g., *Bifidobacterium*, *Lactobacillus*)	Alters the gut microbial composition, leading to a downregulation of the RANKL/OPG ratio to attenuate osteoclast activation, and an upregulation of pro-osteogenic genes like *Bmp-2* and *Sparc*, collectively promoting bone formation.	(66, 68–70)
	3. Lactoferrin modulates gut microbiota to reduce the production of serotonin (5-HT) from tryptophan metabolites.	Inhibition of 5-HT production	Avoids the negative regulation of bone mass by 5-HT, thereby benefiting bone mass maintenance.	(67)
Calcium-Phosphorus Metabolism Regulation	1. Utilizing its mineral-binding properties, lactoferrin forms stable, soluble complexes with calcium (Ca^2+^) and phosphate (PO_4_^3−^) ions in the intestine.	Lactoferrin-mineral complex	Improves the solubility and absorption rate of minerals in the gut, providing a more sufficient supply of raw materials for bone mineralization.	(71)
	2. During fracture healing, lactoferrin enhances the function of osteoblasts.	Enhanced osteoblast function	Promotes new bone formation and mineralization, leading to a shorter fracture healing time and improved bone microstructure (e.g., increased trabecular number and thickness).	(72, 73)

## 6 Clinical applications of lactoferrin in the regulation of bone metabolism

### 6.1 Therapeutic peptides

Lactoferrin is a large molecular protein unfavorable for its effective delivery and realization of biological functions, so lactoferrin-derived peptides have emerged. Lactoferrin-derived peptides can realize a variety of biological functions, such as anti-microbial, anti-inflammatory, and regulation of skeletal cell function (74–76). It has been demonstrated that human lactoferrin-derived peptides can significantly reduce the severity of osteomyelitis in a rabbit model (77). Lactoferrin-derived peptide achieves its function of protecting cartilage by inhibiting IL-1 and fibroblast growth factor 2 and attenuating their degradation of cartilage (78). Lactoferrin-derived peptides also promote the production of the anti-inflammatory cytokine interleukin-11 (IL-11), which in turn stimulates the STAT3 signaling pathway and enhances the expression of TIMP-1 in chondrocytes, which inhibits matrix metallopeptidase 13, a central regulator of chondrocyte senescence (79).

LFP-C and LP2 are lactoferrin-derived peptides that have been shown to have an effect on osteoblast function. LP2 is a peptide derived from human lactoferrin, which has been shown to promote osteogenesis and anti-resorption, and faster fracture healing through activation of p38, MAPK, and increased production of BMP-2 and OPG in ovariectomized (OVX) rats. amino acid peptide derived from lactoferrin conjugation digested by pepsin, which enhances osteoblast differentiation and mineralization as well as the expression of genes involved in bone formation, and subsequently promotes bone formation and mineralization (80). 100 μg/mL of lactoferrin effectively promotes the differentiation of adipose-derived stem cells into osteoblasts through activation of the PI3K/AKT and IGF-R1 pathways, which suggests the potential of LF for clinical use with the OVX rats. LF is promising for clinical use in combination with biomaterials as an innovative molecular and cellular therapy to promote bone repair (81). In conclusion, lactoferrin-derived peptides have strong potential as one of the effective have segments for the treatment of orthopedic-related diseases.

### 6.2 Anti-osteoporosis

Lactoferrin has great potential for the treatment of osteoporosis. It has been demonstrated that after treatment of glucocorticoid osteoporotic rats with lactoferrin, the bone volume, the number of trabeculae, and the thickness of trabeculae of the rats were increased, and the increase increased with the increase of the dose. In addition, lactoferrin inhibits osteoblast apoptosis through autophagy mediated by the AMPK/ULK-1 signaling pathway, thereby exerting an anti-osteoporotic effect (82). A lactoferrin-mimetic peptide, LP2, has been developed, which has shown better results in bone healing compared to standard treatments such as teriparatide (83). In addition, lactoferrin supplements enriched with ribonuclease have been shown to have a positive effect on bone turnover markers in postmenopausal women, indicating reduced bone resorption and enhanced bone formation (84).

In conclusion, lactoferrin holds significant promise as a therapeutic agent for osteoporosis, with the ability to enhance bone formation and inhibit resorption.

### 6.3 Lactoferrin-hydrogel complex slow-release system

Despite its power, it is challenging to precisely achieve an effective bioconcentration of lactoferrin *in vivo* (85). To cope with this problem, lactoferrin-hydrogel sustained-release systems have gradually come into the limelight (86). In a rat femoral defect model species, gelatin hydrogel promoted bone regeneration by releasing lactoferrin. Not only that, there were no signs of inflammation and necrosis around the newborn bone, suggesting that it had a good fusion process with the surrounding bone tissue (87). In addition, chitin/PLGA-CaSO4 hydrogel piggybacked with lactoferrin and substance P was able to significantly promote bone regeneration and new bone formation in a cranial bone defect model, thanks to the fact that the superposition of lactoferrin and substance P enhanced the osteogenic and angiogenic activities of the hydrogel (88). Such studies suggest that lactoferrin can be used in combination with other osteogenic drugs, which in turn produce synergistic effects and enhance the therapeutic efficacy of this system.

Although the lactoferrin-hydrogel sustained-release system demonstrates promising potential for bone regeneration, further research is needed to determine its optimal dosage, release kinetics, long-term biocompatibility, and potential immune responses (89). Additional preclinical and clinical studies should be conducted to evaluate its safety and efficacy (90).

## 7 Discussion

The direct and indirect regulatory effects of lactoferrin on osteoblasts, osteoclasts, osteocytes, and chondrocytes all exert a powerful influence on bone metabolism. Lactoferrin-derived peptides and therapeutic peptide fragments, as the active fragments of lactoferrin, show a great potential in the treatment of orthopedic-related diseases. Meanwhile, the lactoferrin-hydrogel complex extended-release system provides a solution for the precise delivery and maintenance of effective bioconcentration of lactoferrin *in vivo*, further enhancing the therapeutic effects of lactoferrin in bone regeneration and new bone formation. Although significant research progress has been made on the regulatory role of lactoferrin in bone metabolism, many challenges and questions remain to be addressed. For example, the specific mechanism of lactoferrin action *in vivo* still needs further in-depth study, especially its regulatory network and targets of action in different physiological and pathological states. In the future, with the in-depth understanding of the regulatory mechanism of lactoferrin in bone metabolism and the continuous progress of technical means, lactoferrin is expected to play a more important role in the treatment of orthopedic-related diseases. At the same time, the combined application of lactoferrin and other therapeutic means will also become one of the future research directions, in order to realize better therapeutic effects and improve the quality of life of patients.
